# An Empirical Approach for the Development of Process Parameters for Laser Powder Bed Fusion

**DOI:** 10.3390/ma13235400

**Published:** 2020-11-27

**Authors:** Aron Pfaff, Martin Jäcklein, Max Schlager, Wilfried Harwick, Klaus Hoschke, Frank Balle

**Affiliations:** 1 Fraunhofer Institute for High–Speed Dynamics, Ernst-Mach-Institut, Ernst-Zermelo-Str. 4, 79104 Freiburg, Germany; martin.jaecklein@emi.fraunhofer.de (M.J.); max.schlager@emi.fraunhofer.de (M.S.); Wilfried.Harwick@emi.fraunhofer.de (W.H.); klaus.hoschke@emi.fraunhofer.de (K.H.); frank.balle@mail.inatech.uni-freiburg.de (F.B.); 2 Department for Sustainable Systems Engineering (INATECH), Walter and Ingeborg Herrmann Chair for Engineering of Functional Materials (EFM), University of Freiburg, 79085 Freiburg, Germany

**Keywords:** additive manufacturing, selective laser melting, process parameter development, methodology, optimization, new alloys

## Abstract

For certain additive manufacturing technologies the choice of available materials is currently limited. The development of process parameters is especially elaborate for powder bed technologies. Currently, there is no common approach concerning the procedure and documentation. This work proposes a methodology for the initial development of process parameters for new L-PBF (laser powder bed fusion) alloys. Key elements are the examination of the laser–powder-bed interaction by single laser track experiments and an iterative design of experiment (DoE) approach for the development of volumetric parameters. Two types of single laser track experiments are presented and provide information regarding the laser track width and depth as well as the resulting surface roughness and melt pool classification. Based on the information gained, suitable process windows for a DoE study can be defined by avoiding parameter settings unsuitable for production or measurement. Gradually, input variables are identified and iterative steps reduce the process window in order to optimize the desired target values. Near-surface exposure parameters are developed by a one-dimensional parameter variation and metallographic investigations. The approach is primarily designed for the initial development of process parameters for new L-PBF alloys. However, the information gained can also be used to optimize established parameter sets regarding new target values (productivity, mechanical properties), optimize process parameters for specific components or for a microstructural design.

## 1. Introduction

In recent years, additive manufacturing (AM) has developed from a novel prototyping technology towards a multibillion-dollar market. Advantages like no part-specific tooling, customized parts, short lead time, enhanced freedom in design, integral design, integration of function and simplified logistics are some of the key reasons for the steady growth and industrialization of the technology. In particular, metal-based laser powder bed fusion (L-PBF) is being industrialized at a rapid pace due to its high material quality. [1,2] One obstacle for a further increased expansion of the technology is the currently limited amount of available processable materials. Due to the intricate and expensive development of process parameters, the amount of available L-PBF alloys is limited compared to most conventional manufacturing techniques [3]. Aluminum (e.g., AlSi10Mg) and titanium alloys (Ti64) are generally applied for lightweight constructions, nickel-base alloys (e.g., IN718) are suitable for high-temperature applications, stainless steel (e.g., 316L) is used for machine construction and the production of art objects, cobalt, chrome and titanium alloys cover most of the medical technology market and, finally, high-tensile steels (MS1) are applied for the production of tools. Alternative alloys for these fields are scarce and application fields with demand for alternative materials are momentarily only covered to a limited extent. According to Fayazfar [3], other, more adequately tailored AM alloys are needed. Approximately 50 key process parameters need to be defined in order to achieve a material of sufficient quality [4]. Besides the influence on the relative material density, the process parameters also impact the material’s microstructure and, therefore, its mechanical properties [3]. There is currently no uniform approach to this challenge. The development of process parameters is often based on experience and trial-and-error approaches. A standard procedure is also needed for efficient data interpretation and in order to avoid the currently quite common experimental duplication in the AM community [3]. The following work proposes a structured empirical approach for the initial development of L-PBF process parameters, taking the current state of the art into account. The approach can suitably be applied to industrial L-PBF systems, which usually show a limited flexibility regarding the parameter settings.

## 2. State of the Art

The exact amount of process parameters can vary depending on the exact machine setup. Yadroitsev [5] suggests that there are more than 130 parameters that could affect the final material quality. Spears [4] however defines 50 process inputs variables. Some of the latter can consist of several process parameters (e.g., exposure strategy consisting of several geometrical parameter definitions). Furthermore, Spears [4] classifies these 50 process input variables in four categories based on the work of van Elsen [6]: (1) laser and scanning parameters, (2) powder material properties, (3) powder bed properties and recoat parameters, and (4) build environment parameters. Spears [4] also distinguishes between “predefined” and “controlled” parameters. However, the sources above agree that the most significant parameters include laser power, scanning speed, hatching (laser-track spacing), layer thickness, scanning strategy, atmosphere and powder bed properties. An enhanced factor analysis is presented by Yadroitsev [7] with respect to the importance of single process parameters. Most publications regarding the development of new process parameters seem to focus on laser power, scanning speed and hatching.

Considering the number of parameters, design of experiment (DoE) is a common approach in order to identify optimal process parameters [8]. Examples are shown by Kamath [8], Delgado [9], van Elsen [6] and Enneti [10]. Another common method is the use of the volume energy density (*VED*) [11]:

(1) $$VED_{\mathrm{laser}}=\frac{P}{vtd}\;\left[\frac{J}{{\mathrm{mm}}^{3}}\right]$$

where *P* is the laser power, *v* the scanning speed, *t* the layer thickness and *d* the laser beam diameter. An alternative approach is given in [12], where the laser beam diameter is replaced by the hatch distance h:

(2) $$VED_{\mathrm{hatch}}=\frac{P}{vth}\;\left[\frac{J}{{\mathrm{mm}}^{3}}\right]$$

A list of studies using these approaches can be found in [3,13,14,15]. However, recent studies envisage certain restrictions regarding this equation. As a thermodynamic quantity, *VED* is not able to describe complex phenomena like recoil pressure, hydrodynamic instabilities or the Marangoni flow. Heat and mass transportation are, therefore, not considered according to Bertoli [11]. Furthermore, the *VED* approach does not consider further process parameters to be of importance concerning the actual local energy density, like the hatch spacing/laser beam diameter, exposure strategy, inert gas flow or laser offsets [12]. While Bertoli [11] proves the limitations of *VED* approaches by investigating single laser tracks, Prashanth [12] demonstrates that a constant *VED* can cause different material properties with respect to density and the resulting quasi-static stress–strain curve based on the chosen process parameters.

Many studies investigate the melt pool characteristics regarding the melt pool width (*W*) and depth (*D*). A common approach as shown by Dilip [16], Gong [17], Pfaff [18], Kempen [19], Wits [20], Yadroitsev [7] and Sadowski [21] is to apply a single laser track per inquired parameter combination of laser power and exposure speed directly onto a bulk material consisting of the investigated alloy (bulk approach). Alternatively, single laser tracks can be applied repeatedly in order to create a thin-walled structure, which can be used to determine the fuse width (tin-like-structure approach). Xiong [22] uses this approach by creating rectangular structures consisting of a single laser track line. Based on a microscopic analysis, Xiong classifies three different groups: excessive melting, good melting and weak sintering. Similar visual classifications can be found in Kempen [23], Gong [17] and Yadroitsev [7]. The visual investigation of single laser tracks usually serves as a basis for the definition of an adequate process window concerning the correlation of laser power and exposure speed. Seede [24] also uses the melt pool dimensions to divine a maximal hatch spacing under the assumption of a parabolic melt pool geometry:

(3) $$h_{\max}=W\sqrt{1-\frac{t}{\left(t+D\right)}}\;\left[\mathrm{mm}\right]$$

The experimental proceeding regarding the investigation of a solid material consisting of an arrangement of laser tracks and, therefore, an increased parameter amount shows a variety of approaches. L-PBF’s typical defects have to be considered. The most dominant defect modes are lack-of-fusion (LOF), balling, and keyhole formation. Keyhole-mode laser melting, for example, causes evaporation due to the power density. This evaporation causes a cavity that increases laser absorption (e.g., by multiple reflections), resulting in a deeper melt pool than possible by a purely conduction-driven mode. As shown by Madison [25], characteristic voids within the laser track are the result. LOF results from insufficient penetration into the substrate, causing large and sharp pores, while balling describes a periodic oscillation of the width of the melt pool tracks [3,26]. In literature, the extent of these effects is often described by defining four different categories resulting from the layer thickness (*t*), melt pool width (*W*), depth (*D*) and length (*L*). While a ratio *D/t* < *X1* is defined as an area of LOF, *L/W* > *X2* represents balling phenomena and *W/D* < *X3* areas with “key holing” effects. Parameter combinations falling under none of these definitions are assigned a suitable category. The threshold (*X1, X2, X3*) of the presented ratios has to be calibrated based on the boundary conditions like used machine type or alloy [26,27,28,29].

Regarding the order of parameter definition, Letenneur [30] proposes to first estimate a layer thickness, followed by the determination of a maximum hatch distance for an increased productivity. A *VED*-build-rate diagram is used in order to define the required *VED* for a maximum density. Hatch distance and *VED*-build-rate diagram are based on analytical solutions. In addition, the arrangement of the laser tracks within one layer is of high importance as shown by Rashid [31]. Considering that a double exposure can be of advantage for certain alloys, the range of possible laser track arrangements is immense.

Recent approaches combine the simulation of single laser tracks including an experimental validation. Concerning the process simulation for solid material, three kinds of approach can be found in literature: numerical, analytical and empirical [3]. However, process simulations for the L-PBF process are complex, since special effects like the interaction between metallic powder particles and the laser beam are currently not fully understood as shown by Foroozmehr [32].

Johnson [26] presents an approach in order to achieve two-dimensional printability or process parameter maps based on laser power and exposure speed by defining *D/t* < 1.5, *L/W* > 2.3 and *W/D* < 1.5. Johnson [26] uses numerical approaches in order to quantify *D*, *W* and *L* (Finite Element and Eagar–Tsai model). The validation shows deviations especially regarding the melt pool depth. Seede [24] enhances the approach presented by Johnson [26] by adding the maximum hatch distance as a third dimension to the printability map. For the process parameter development for a martensitic steel, Seede [24] uses a comprehensive approach using FE simulation in order to predict the geometrical melt pool characteristics. Single track experiments are used for calibration of the simulation in order to achieve a precise melt pool prediction. The hatch space is defined by Equation (3) and the processing map is class-divided by the following criteria: *t* < 1 (LOF); *D* < *W*/1.2; while the balling area was defined by experimental investigations.

The development of process parameters is often based on an optimization of the material’s relative density. However, avoiding porosities is not necessarily the only purpose of choosing a process parameter set. Also, the microstructure can be impacted [33]. Therefore, it can be advisable to optimize the manufacturing parameters considering multiple requirements as shown by Xiong [22], who is assessing the process parameters regarding the resulting relative density and hardness. In addition, knowledge of the impact of different process parameters enables the manufacturing of a locally structured material or designed microstructures, as shown by Pfaff and Popovich [18,33,34,35]. Apart from the specific studies above, there are currently no available approaches which integrate the single experimental steps to a systematic approach for the development of process parameters.

## 3. Methodology

### 3.1. Basic Determinations

Based on the specific studies above, a universal approach for the development of the L-PBF process parameters is developed here with the following considerations:

Regarding process parameter classification: the classifications (1) laser and scanning parameters, (2) powder material properties, (3) powder bed properties and recoat parameters, and (4) build environment parameters proposed by Elsen, Spears and Mani [4,6,36] are applied in this work. Additionally, for a more practical use, parameters designated as “controlled” are differentiated into the groups “controlled (per specimen)” and “controlled (per build)”, while “predefined” ones are subdivided into “predefined (machine setup)” and “predefined (used material)”.

Regarding *VED* and process simulations: considering the studies of Bertoli [11] and Prashanth [12], the *VED* seems to be a limited parameter and will, therefore, not be used in this approach. Currently the use of numerical simulations shows certain challenges. The result needs to be controlled by practical investigations. Validated simulations are also only valid for a single experimental condition (e.g., type of L-PBF machine, alloy environmental conditions). Therefore, an experimental approach is always necessary and can currently not be replaced by simulation [3]. Alternatively, neural networks could be a promising approach. However, the database to train a reliable neural network currently seems too small. Furthermore, published results are usually incomplete with respect to the applied process parameters. However, complete sets would be needed to build a thorough database. The presented work is therefore an empirical approach, while certain steps could be replaced by future reliable simulations to minimize the amount of practical experiments.

**Figure 1:**
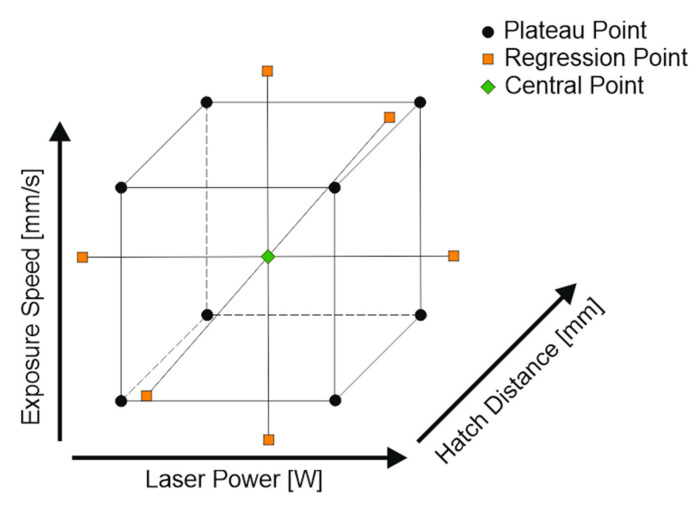
Schematic representation of the applied design plans for a 3-dimensional central composite design (CCC, three continuously variable input parameters) with α = √2.

Regarding experimental plans: as postulated by Fayazfar [3], the presented statistical approach includes a consideration of higher order interactions while conducting a minimum of experiments due to the extensive amount of process parameters. For this reason, DoE is used in this approach. Figure 1 shows a schematic representation of the applied central composite design (CCC), an extended form of the full factorial design, for a 3-dimensional design (three continuously variable input parameters). Siebertz [37] provides further information regarding the chosen DoE design and the equations used.

A characteristic property of the statistical experimental design is the choice of test points based on parameter levels. By comparing levels using *t* and *F* tests, statements about the significance of a parameter change can be made without performing repeated measurements for the same experimental point that would be needed to obtain a statement about the standard deviation. The *t*- and *F*-values are defined as follows:

(4) $$t=\frac{{\bar{X}}_{1}-{\bar{X}}_{2}}{s_{p}\cdot \sqrt{\frac{1}{n_{1}}+\frac{1}{n_{2}}}}$$

(5) $$s_{p}=\sqrt{\frac{\left(n_{1}-1\right)s_{1}^{2}+\left(n_{2}-1\right)s_{2}^{2}}{n_{1}+n_{2}-2}}$$

(6) $$F=\frac{s_{x_{1}}^{2}}{s_{x_{2}}^{2}};F\geq 1$$

By introducing a central point (see Figure 1), it is possible to efficiently determine whether a significant effect of a parameter change has a linear correlation. If no linear correlation can be verified, the introduction of regression points (see Figure 1) allows quadratic regressions. Based on the data points obtained and regression methods, a model equation can be derived for the n-dimensional parameter space:

(7) $$\tilde{\eta }\left(x,\vartheta \right)=\vartheta_{0}x_{0}+\overset{n}{\underset{i=1}{\sum }}\vartheta_{i}x_{i}+\overset{n}{\underset{i=1}{\sum }}\vartheta_{\mathrm{ii}}x_{i}^{2}+\overset{n}{\underset{\begin{array}{c} i=1\\ j=2,i<j \end{array}}{\sum }}\vartheta_{\mathrm{ij}}x_{i}x_{j}$$

This allows, for example, the visualization of the correlation between two parameters by generating topographic diagrams and the representation of main factors and interactions. The quality of the generated model equation is quantified with the aid of the coefficient of determination:

(8) $$R^{2}=\frac{\sum_{i=1}^{n}{\left({\hat{y}}_{i}-\bar{y}\right)}^{2}}{\sum_{i=1}^{n}{\left(y_{i}-\bar{y}\right)}^{2}}$$

It should be noted that this form of statistical design is only suitable for normally distributed samples due to the use of *t*- and *F*-tests. Investigations on previous L-PBF materials show a standard distribution. Regarding density measurements however, this assumption cannot be retained for samples with a relative density close to 100%.

**Figure 2:**
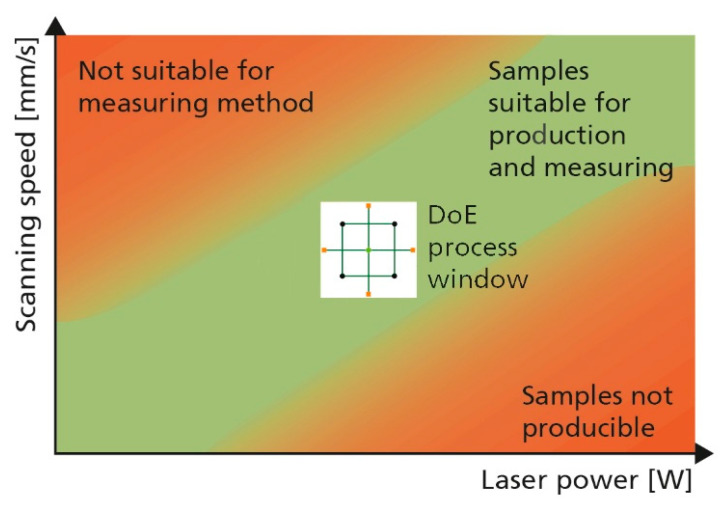
When choosing the test points (which define the process window) for a statistical experimental design for L-PBF (laser powder bed fusion) it is crucial to avoid areas causing faulty manufacturing or resulting in specimens unsuitable for the intended form of measurement.

Decisive for the quality of a model equation generated by statistical experimental design are exact and reproducible measurements [37]. For this purpose, it is necessary to produce samples of sufficient quality for the method of measurement used. If, for example, the density is investigated based on the Archimedes principle, an increased porosity resulting from too low an energy input into the powder bed leads to distorted measurements due to escaping gases during the density measurement. Excessive energy input, on the other hand, can lead to overgrowths in form of material agglomerations, which lead to jamming of the recoater system and thus to a faulty or even aborted L-PBF process. It is important to avoid these extremes when choosing the process window for the statistical experimental design. In particular, considering that DoE approaches exhibit test points positioned in the extremes of the regarded process window, the test points of the experimental plans should lie between the mentioned extreme ranges. Figure 2 presents an example based on laser power and exposure speed. The regarded process window should be within the range of suitable specimens.

### 3.2. General Workflow

**Figure 3:**
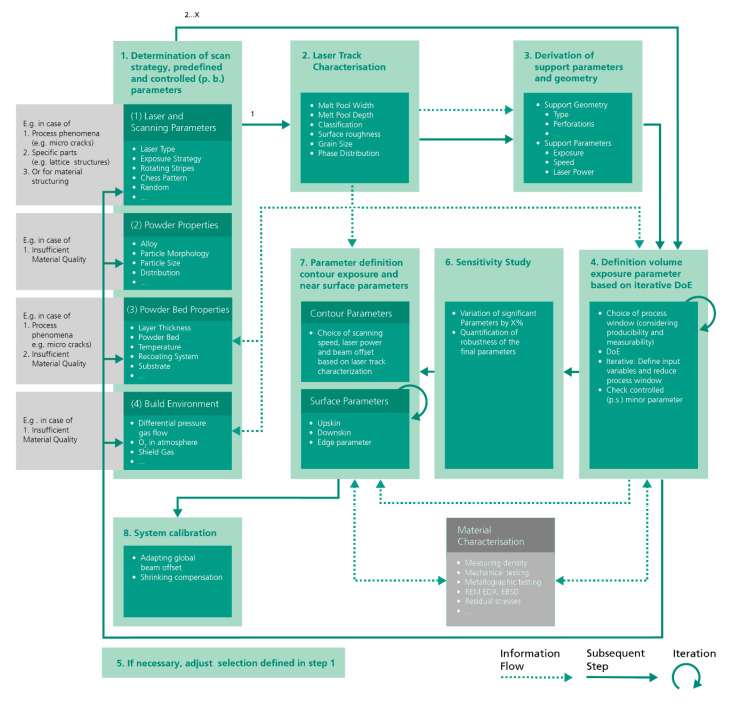
Schematic design of the proposed approach.

The approach consists of eight fundamental work steps (see Figure 3):

1. Determination of scan strategy, predefined and controlled (per built) parameters;
2. Laser track characterization;
3. Derivation of support parameters and geometry;
4. Definition of volume exposure parameters (by iterative DoE);
5. If necessary, adjustment of the selection defined in step 1;
6. Sensitivity study;
7. Definition of contour exposure and near surface parameters;
8. System calibration and compensation of shrink factors.

### 3.3. Single Workflow Steps

The following section presents the structure of the single work steps. Material related results shown in tables, graphs and figures are based on the alloy MS1 (1.2709 / X3NiCoMoTi 18-9-5), processed on an EOS M 100 L-PBF machine in 30 µm layers. The approach has been developed and successfully tested based on the materials listed in Table 1.

#### 3.3.1. Step 1: Determination of Scan Strategy, Predefined and Controlled (Per Built) Parameters

All predefined parameters show a limited flexibility and are, consequently, less suitable for a variation within an efficient systematic investigation. Therefore, these parameters are defined from the start, with respect to the state of the art and experience. Some examples of these more rigid parameters are the used laser type, substrate material, powder material or oxygen concentration. Overall, changing the L-PBF machine setup or the powder material is time consuming and difficult and should, therefore, be avoided if possible. Also, process parameters of the type “controlled (per build)” are predefined in this step since these values can usually easily be chosen based on experience and literature. The reduced parameter space simplifies the experimental design.

In contrast, the scanning strategy represents an exception within this step. Theoretically, the geometrical arrangement of laser tracks within one layer is extremely flexible and can be changed within one build (controlled (p. b.)). However, only a certain amount of well-known scanning strategies is proven appropriate. Therefore, a prevalent strategy should be defined while keeping the geometrical parameters like beam offset (BO) values undefined. This example shows a stripe strategy with a contour exposure line and adapted parameter for near surface areas in order to consider changing thermodynamic boundary conditions (e.g., minor heat flow due to an increased amount of unmolten powder particle in proximity resulting for example from flat angle surfaces). The choice of the general scanning strategy will result in the definition of several geometrical parameters for different exposure areas, which will be defined in steps 4 and 6, e.g.: stripe width, stripe overlap, rotation angle, single exposure, BO.

A full example of the predefined parameters and the definition of the scanning strategy resulting from this step is shown in Table 2. The number of listed parameters depends on the available information, kind of investigation, used methods and available data. The type definition depends on the L-PBF machine utilized and software tools.

Additional information regarding step 1:

When selecting the substrate material, attention must be paid to the surface roughness, weldability and to similar coefficients of thermal expansion in the material combination. In general, construction platforms made of the alloy of the powder material would be well suited, but cannot always be realized, for example, for financial and manufacturing reasons. The determination of oxygen concentration, platform temperature, exposure strategy and choice of recoating system and layer thickness are based on experience with similar alloys and literature research (e.g., necessary conditions for oxide formation, reactivity, heat treatment of materials). The powder is chosen on account of the selected layer thickness as well as empirical values based on other metal powders already processed in the L-PBF procedure. In general, a high tap density and a distinct flowability is beneficial. Spherical morphology of the powder particles favors these factors [39].

#### 3.3.2. Step 2: Laser Track Characterization

**Figure 4:**
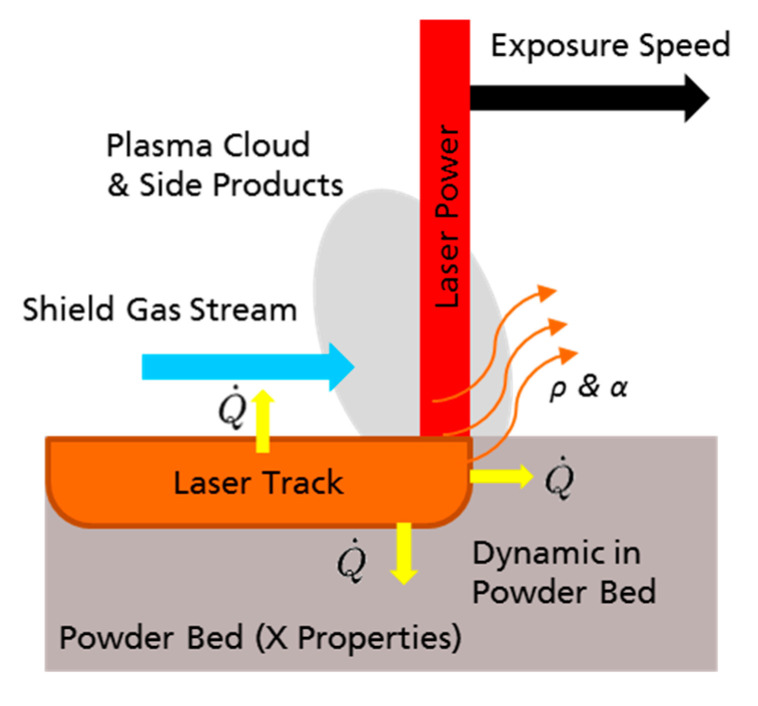
Parameters like absorption/reflection, heat flows, plasma cloud in combination with scan speed and side products influencing the interaction between laser beam and powder bed. $\dot{Q}$: Heat flow; *ρ* reflectance; α: absorbance.

The first experimental step is to characterize the result of the interaction between laser beam and powder bed and consists of single laser track experiments. The approach does not consider the complex physical interactions depending on several phenomena and parameters (see Figure 4), but only characterizes the relevant resulting melt track.

**Figure 5:**
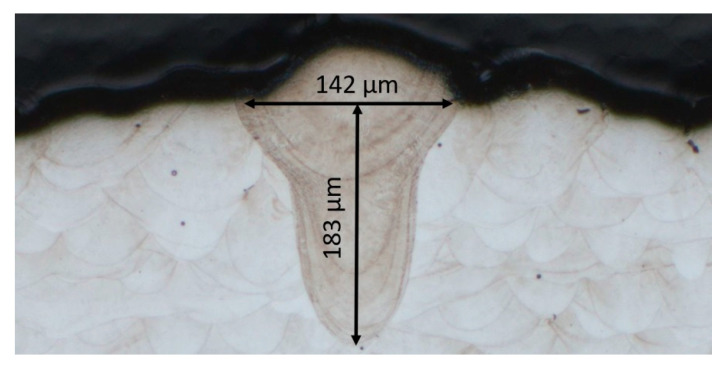
Single laser track of 150 W and 800 mm/s applied onto a bulk material showing the measured width (*W*) and depth (*D*) by optical microscopy. Since no conventionally manufactured MS1 bulk material was available for this work, tin-like-structures (see Figure 6) were used for the development of the process parameters. The results were compared to the bulk material approach after the development of suitable process parameters, based on a bulk material manufactured by L-PBF.

These experiments exist in different designs. A common approach is the application of single laser tracks directly on a bulk material consisting of the investigated alloy or onto a single powder layer. A result of this approach is shown in Figure 5. However, a bulk material of the same composition as the metallic powder is not always available and exhibits different thermal boundary conditions than practical cases. Also, the application of a single and precise powder layer is intricate.

**Figure 6:**
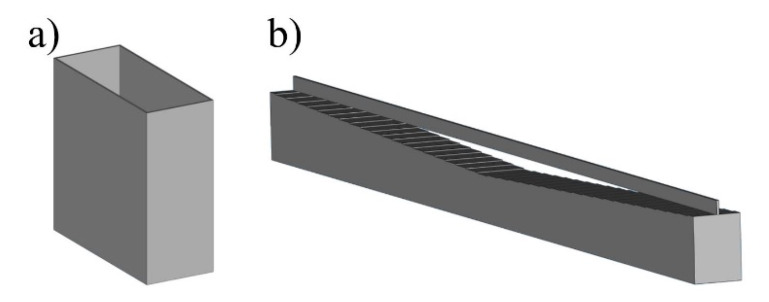
Specimen for the quantification of laser track width and penetration depth based on tin-like-structures. Structures consisting of a single laser track. (**a**) Width. (**b**) Depth.

In order to avoid some of the challenges described above and also to simplify the characterization process, an approach as shown by Xiong [22] can be used as an alternative. In order to quantify the laser track width, a closed laser track geometry is applied over several layers resulting in a tin-like-structure (see Figure 6a) which can easily and efficiently be characterized by light microscopy or by tactile measuring tools. Based on the surface structure (porosity and surface roughness) of the tin-like-structure, statements can be made with regard to the melt track stability.

Compared to the bulk approach, local inaccuracies in the laser track and distorted results due to changing conditions (e.g., irregular thickness in powder layer) can be avoided. An alternative way to quantify the cure depth is shown in Figure 6b). First, a step-shaped specimen is generated. One step equals one layer thickness. At the end of the specimen, a single laser path with defined parameters is drawn over the body. By investigating the steps connected with the laser track, it is possible to deduce the penetration depth.

**Figure 7:**
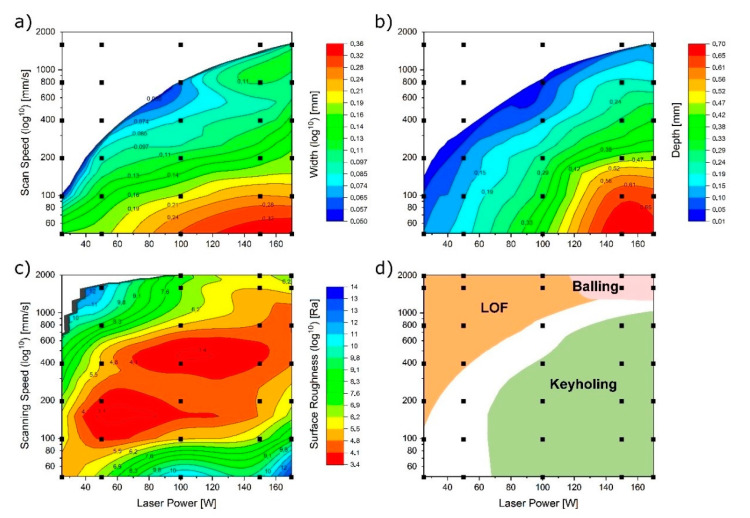
Exemplary resulting information of step 2 for MS1 (*t* = 30 µm). Black squares mark points of measurement and interpolation. (**a**) Fuse width. (**b**) Fuse depth. (**c**) Surface roughness. (**d**) Printability map. (*D/t* < 1.5, *L/W* > 2.3 and *W/D* < 1.5).

The findings regarding the melt track stability classifications and surface roughness support the choice of a suitable parameter window for the DoE approach as well as the choice of suitable contour parameter. The geometrical information on the other hand supports the choice of laser track distances (e.g., hatch, BO) and a suitable combination of layer thickness and exposure parameters. The findings are incorporated in all subsequent steps of the parameter development. An exemplary result of this step is shown in Figure 7.

The advantage of this approach is that the impact of several currently difficult-to-determine parameters and their unknown interactions are recorded by relevant and quantifiable measures. For example, the influence of the thermal conductivity of the powder bed, enthalpy of fusion or the heat flow induced by the inert gas flow is considered in the measurement result (see Figure 4). In this way, currently unreliable process simulations are bypassed. Additionally, the first observations and adaptions regarding parameters of the group’s (3) powder bed properties and recoat parameters and (4) build environment parameters can be made (e.g., suitability of substrate, adapting shield gas flowrate, recoating speed). The results of this step strongly depend on the thermal conductivity of the regarded alloy. Especially the melt pool width but also the surface roughness increases with an increasing thermal conductivity. Also, the energy coupling into the powder bed and, therefore, the laser caustic and intensity profile are major impact factors. Experiments executed too far out of the focus layer can show strong deviations. This effect seems to be more pronounced for systems with a poor laser caustic and therefore beam quality M^2^.

#### 3.3.3. Step 3: Derivation of Support Parameters and Geometry

The use of support structures provides an ergonomic work flow, since specimens in following steps can easily be removed by hand from the substrate instead of using time-consuming processing by machine. The relevance for the material quality of the support part can be neglected. This step, therefore, serves as a measure to accelerate later experimental studies on volumetric material. Parameters showing high energy density but no excessive melting ensure a sufficient melting of the material and are, therefore, advisable for support structures. The geometrical design can be designed on experience with similar alloys and improved in the following process in case of defects.

#### 3.3.4. Step 4: Definition Volume Exposure Parameter (by Iterative DoE)

Based on the single laser track experiments, suitable volume exposure parameters are developed within this step. This process is subdivided into the following sub-steps:

1. Choice of process window based on suitable major controlled (p.s.) main parameters: laser power, scanning speed and hatching.
2. DoE: Central Composite Design (CCC).
3. Limit variables and reduce process window.
4. Check controlled (p.s.) minor parameter.

The first investigations within this iterative step assess the controlled (p.s.) main parameters laser power, scanning speed and hatching, while keeping minor parameters constant. The limits of exposure speed and laser power can be deduced from the observed stability of the melt track as well as the definition of *D/t*, *L/W* and *W/D* in order to avoid LOF, keyhole formation and balling phenomena. In this work, the following values defined by Johnson [26] are applied: *D/t* < 1.5, *L/W* > 2.3 and *W/D* < 1.5.

The choice of the hatch distance is based on the selected exposure speed and laser power. In order to validate Seede’s [24] analytical approach for the determination of the hatch distance (*h*), Equation (3) was applied for the following already existing L-PBF alloys and corresponding process parameters resulting in high densities: Ti64 (*t*: 20 and 30 µm), MS1 (*t*: 30 and 60 µm), AlSi10Mg (*t*: 30 and 60 and 90 µm). Using the melt pool width quantified by single laser tracks on a bulk material followed by a microscopic visual analysis results in a deviation of ~66% (σ = 12) between the used and calculated hatch distance. Using data generated by tactile measurements of tin-like structures (representing the maximum width, including surface agglomerations) results in a deviation of ~120% (σ = 11). While the maximal hatch distance can theoretically be estimated by Equation (3), in reality the laser tracks might exhibit local irregularities resulting in porosities. Therefore, a safety factor (*s*) is introduced in order to compensate for irregularities. Based on the analysis above, *s* = 0.65 using a database generated by the bulk approach and *s* = 0.5 for the tin-like structures approach. The hatch distance (*h*) can, therefore, be defined based on the safety factor (*s*), melt pool width (*W*), depth (*D*) and layer thickness (*t*):

(9) $$h=sW\sqrt{1-\frac{t}{\left(t+sD\right)}}\left[\mathrm{mm}\right]$$

**Figure 8:**
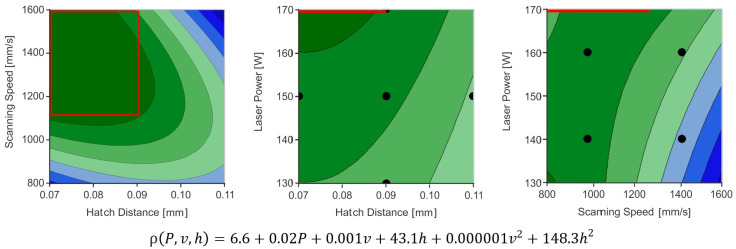
Example of a resulting model equation and its partial visualization (hold values: *P* = 170 W; *v* = 1200 mm/s; *h* = 0.1 mm). For an increased productivity, the hatch distance can be set to 0.1 mm, since the value still allows a high density. The chosen process window for the second iteration is marked in red. Black marks indicate points of measurement. Below: model equation (*R2* = 95.7).

The hatch distance and further processing parameter for the materials above cannot be published within this document for legal reasons. The maximum and minimum of the calculated hatch distances and the corresponding exposure speed and laser power are used for a 3-dimensional CCC design. Figure 8 illustrates an example. It is advisable to check the measurements for normal distribution for example by a chi-square- or a Kolmogorov–Smirnov test.

After the evaluation of the first process window, the input variables can be reduced and the parameter window further restricted. For this, hold values are defined for insignificant or less significant parameters. Figure 8 presents an example, where the laser power results in a minor linear increase in density (see model equation). Also, parameters with pronounced maxima under consideration of the interactions can be defined. Therefore, a hold value at the maximum laser power (170 W) is defined and a new process window for the second iteration is selected (see Figure 8 left graph). Subsequently, the considered process window can be further restricted or moved. When defining individual parameters and redefining process windows, other relevant factors such as the productivity of the process can be considered. Since linear relations result in maxima at the edge of the process window, a shift of the process window can be beneficial. Fundamentally, an evaluation of the considered process window by determining the maximum of the model equation would be possible. However, this is not always advisable due to quadratic regression and inter- and extrapolation in peripheral areas. The reason is that the maxima are often located in the peripheral regions and, thus, in the least accurate areas of the process window due to extrapolation. It should be noted that the result of the DoE approach is presenting a quadratic representation and, therefore, a simplification of the actual correlations. Therefore, it can be misleading for certain correlations like functions of third or higher order, jump functions, progressive or degressive curves.

**Figure 9:**
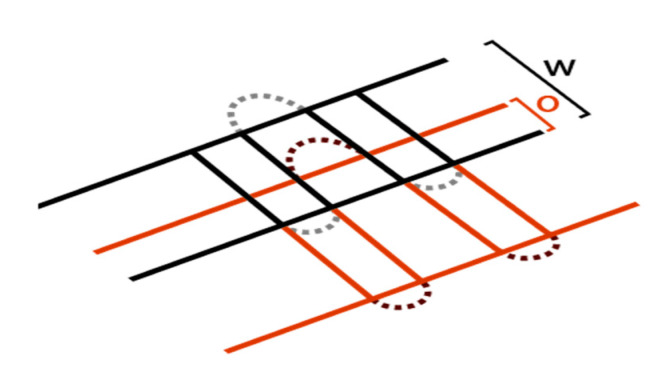
Example of controlled (p.s.) minor parameters like rotation angle, stripe width (*W*), overlap (*O*) or beam offset depend on the chosen exposure strategy (here: stripe exposure type) and should be investigated on their impact.

If an optimum is found through the variation of the controlled (p.s.) main parameters, the controlled (p.s.) minor parameters are investigated in order to increase the target value or to ensure a stable process parameter set. This can be done by the same approach as above. The amount and kind of minor parameters depend on the chosen exposure strategy (e.g., stripe width, stripe overlap; see Figure 9).

Additional information regarding step 4:

Furthermore, during parameter development, attention should be paid to sample positioning in the powder bed. Inhomogeneous heat flows can, for example, result from varying properties of the shielding gas flow and thus influence the manufacturing result. For this reason, it is advisable to initially position samples centrally, avoiding placing them close to the inlet or outlet of the protective gas flow. The position of different specimens relative to each other should also be considered. Too many consecutive samples in the recoating direction could lead to a successive reduction of certain powder particles and thus to different bulk densities. In addition, in the direction of the shielding gas flow, attention should be paid to an appropriate arrangement so that the by-products caused by welding of a certain processing area do not affect adjacent samples. Additionally, the exposure direction should be executed against the gas flow. In order to generate all samples of a test series under similar boundary conditions, it is recommended to produce all samples of a test series in a single production run. If this is not possible, blocked experimental designs should be used. Furthermore, due to the positioning effects mentioned above, randomization should not only be performed in the course of the measurements, but regarding the sample arrangement to identify systematic distortion due to positioning in the powder bed or the order of measurement.

#### 3.3.5. Step 5: If Necessary, Adjust the Selection Defined in Step 1

Certain process phenomena can lead to the circumstance that the target value is limited in its maximum (e.g., limited suitability of powder for process, formation of micro cracks). Depending on the observed defect (e.g., by metallographic methods) countermeasures can be taken by adapting parameters with respect to (1) the laser scanning parameters, (2) powder properties, (3) powder bed properties or (4) build environment.

#### 3.3.6. Step 6: Sensitivity Study

Defects, pollution or other disturbances within the L-PBF process can lead to varying process parameters and therefore resulting material qualities. Possible examples are polluted optical units (e.g., locally contaminated protection glass due to handling errors between builds), aging of the laser unit, or recoating errors. It is advisable to check the defined process parameter regarding their robustness. Possible process parameter deviations need to be defined depending on the material used and L-PBF system (e.g., assumption of 5% deviation in laser power due to locally contaminated protection glass). A CCC design or Taguchi design based on a variation of the presumed parameter variation is used to check whether the variation results in any significant change of the desired material properties.

#### 3.3.7. Step 7: Definition of Contour Exposure and Near-Surface Parameters

In order to achieve an improved surface quality, contour and near-surface exposure parameters can be applied. BO values can be defined by Equation (9). Areas with a distinct pointed angle between substrate and surface exhibit decreased cooling rates, since the increased amount of powder and, therefore, gas in the surrounding volume has an insulating effect (downskin areas). In order to avoid defects or uncontrolled melting and, therefore, poor surface qualities in these areas, exposure parameters consisting of a lower *VED* are used. The analysis of existing process parameters shows a reduced *VED* of 11–72%. The value seems to depend strongly on the processed material. In this approach, it is advised to systematically increase the scanning speed by 10% while adapting the hatch distance based on Equation (9) and keeping other parameters like laser power constant compared to the volume parameters of step 4.

Regarding contour exposures in order to improve the surface quality of L-PBF parts, suitable laser powers and scanning speeds are based on the surface roughness and melt pool classifications of step 2. In general, a higher energy input leads to a lower surface roughness but also to a lower shape tolerance. It has to be considered that the result might vary depending on geometry for thermal reasons and whether the exposed area is in a downskin area or beyond. Therefore, it is recommended to develop the contour exposure together with other near-surface parameters.

**Figure 10:**
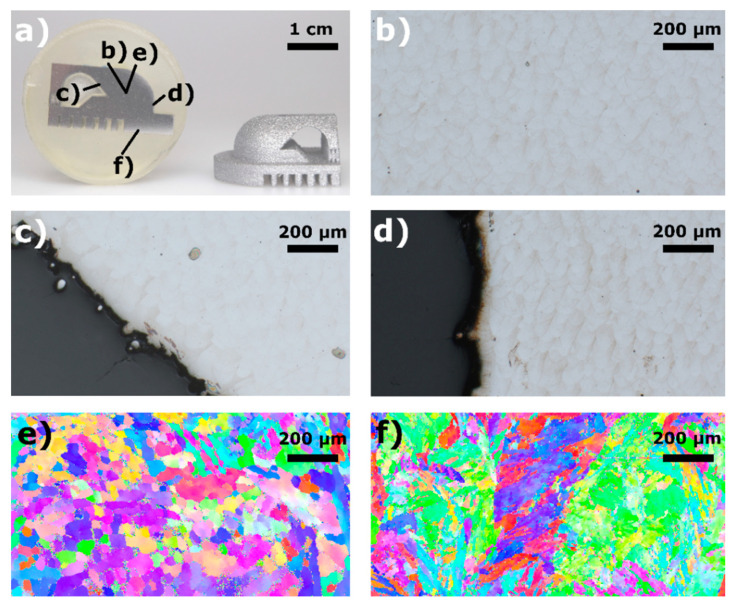
(**a**) Benchmark in its raw form and as a metallographic specimen. (**b**) Microstructure resulting from the volumetric parameters developed in step 4, serving as a reference. (**c**) Microstructure resulting from downskin exposure parameters. (**d**) Areas resulting from contour exposure. (**e**,**f**) Electron backscatter diffraction (EBSD) can be used to evaluate microstructural changes due to changing exposure parameters in near surface areas.

The results can be validated on benchmark parts suitable for near surface parameters as presented by van Elsen [6] or shown in Figure 10. Measurements allowing local evaluations like microscopy or electron backscatter diffraction (EBSD) are applied in order to analyze the resulting density, microstructure and homogeneity. First, a reference analysis is executed in an area manufactured by the volumetric parameters developed in step 4. Near-surface areas based on an adapted scanning speed and hatch distance are, afterwards, compared to the reference analysis (see Figure 10). When observing near-surface porosity, the geometry of the porosity can indicate the reason for the porosity as shown by Madison and Kasperovich [25,40]. The finding can be used to deduce possible causes and to derive corresponding countermeasures.

The results in Figure 10 show a successful adaption of the down- (Figure 10c) and contour- (Figure 10d) exposure parameters regarding the resulting porosity in comparison to the central reference point Figure 10b. The EBSD mapping shown in Figure 10e represents a measurement at the central reference point Figure 10b. An additional EBSD mapping Figure 10f in a downskin area shows a significant change in microstructure. The grain sizes increase significantly and seem to reveal preferential directions in comparison to a central point as shown in Figure 10e. It is unknown if this change in microstructure results from the application of adapted exposure parameters or from the impact of a change in heat flows due to an insulating layer of powder below the downskin area. The observed microstructure could possibly lead to a decreased tensile strength and increased ductility in the downskin area.

#### 3.3.8. Step 8: System Calibration and Compensation Shrink Factors

Finally, a system calibration and compensation of the shrinkage factors should be carried out, for example according to the specifications of the system manufacturer in order to generate geometrically precise components.

### 3.4. Validation of the Resulting Material Quality

**Figure 11:**
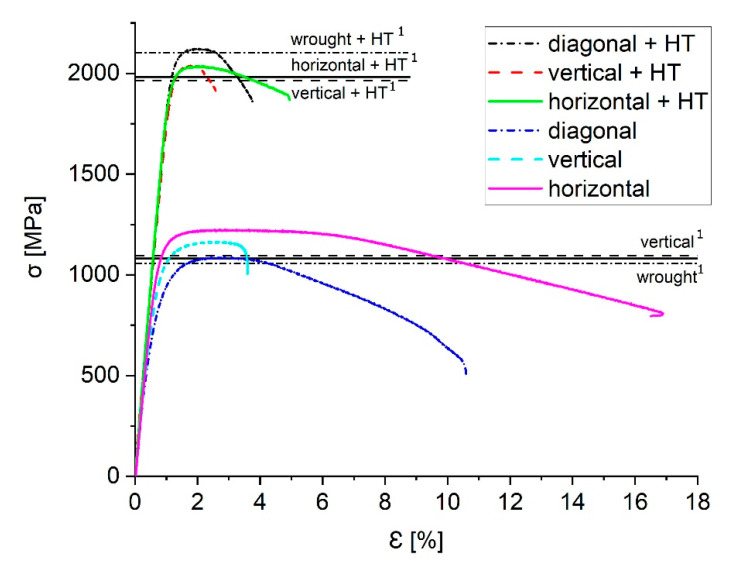
Tensile tests comparing the resulting material quality with literature values presented by Simson [43] for L-PBF- and wrought material. ^1^ Literature values presented by Simson [43].

The approach above is presented based on the example of the parameter development for MS1 (1.2709 / X3NiCoMoTi 18-9-5). Tensile tests executed on “heat-treated” samples (HT; 490 °C 6 h) and “as-built” samples demonstrate the resulting material quality. The heat treatment of a maraging steel is leading to the formation of intermetallic precipitations. The microstructure develops carbide precipitation along the grain boundaries. These microstructural changes lead to a strong increase in strength but also a reduction in ductility [41]. “Heat-treated” samples as well as “as built” samples are tested in the orientations 0° (vertical), 45° (diagonal) and 90° (horizontal), in order to quantify the resulting anisotropy. The average density of the samples is 8.0612 g/cm^3^ (relative density: 99.52%, σ = 0.048). Machined surfaces avoid any impact of the rough L-PBF surface. Each material state is tested three times according to DIN EN ISO 6892 [42]. Tests are executed on an Instron 8033, while the strain is measured by a tactile extensometer. Figure 11 shows one representative curve for each material state. As comparison values, the horizontal lines in Figure 11 mark the ultimate tensile strength (UTS) for L-PBF- and wrought-material presented by Simson [43]. Wrought material serves as a good reference material, since it typically shows a low porosity and high tensile strength.

The vertical and horizontal “as built” samples exhibit an increased UTS in comparison to common values as presented by Simson [43]. The diagonal samples show similar values to the literature. For the HT state, all samples exhibit increased UTS in comparison to the L-PBF material. However, only the diagonal state can match the value of a wrought + HT material. It has to be mentioned that the vertical samples show a decreased breaking elongation, while the diagonal and horizontal samples possess a similar breaking elongation in comparison to the literature values. In general, the results show a distinct anisotropy. Future approaches could include strategy to avoid unwanted effects like anisotropy, since these effects are not considered in the presented approach.

## 4. Extended Fields of Application

Above, the application of the approach is demonstrated for an **initial parameter development** for the alloy MS1 (1.2709 / X3NiCoMoTi 18-9-5). However, the data resulting from the approach can also be used in order to **optimize existing standard parameters**, for example in order to achieve higher productivity under the consideration of new target values concerning the material properties. Therefore, the model equation of the regarded process windows in step 4 can be re-evaluated with respect to different target values. In order to adapt the proportion of material strength and ductility, the established process windows can be re-evaluated regarding the resulting microstructure.

An additional metallographic analysis of the single laser track experiments (step 2) provides information concerning the resulting grain sizes and phase precipitations. This knowledge can support the development of **designed microstructures** as shown in [18,33,34,35] or in order to adapt the proportion of material strength and ductility. However, these microstructural results can only be seen as a trend, since the application of a volumetric scanning strategy will result in different thermal boundary conditions and an in situ heat treatment within the process.

Considering the unique form of each producible component, it can also be of advantage to **optimize process parameters for specific components** in order to increase the resulting quality (e.g., preventing defects by adapting the process parameters to local requirements) or decreasing the process time. Geometries like lattice structures resulting in small cross section areas for example, exhibit shorter laser tracks than usual geometries. Inertia effects like the laser activation time or acceleration phases of the mirror units can lead to local inaccuracies in these geometries. The shortened laser tracks also lead to an increased temperature due to an increased exposure rate and, therefore, to possible defects. The process maps of step 2 and 4 can support the finding of suitable parameters for these cases.

## 5. Conclusions

**Figure 12:**
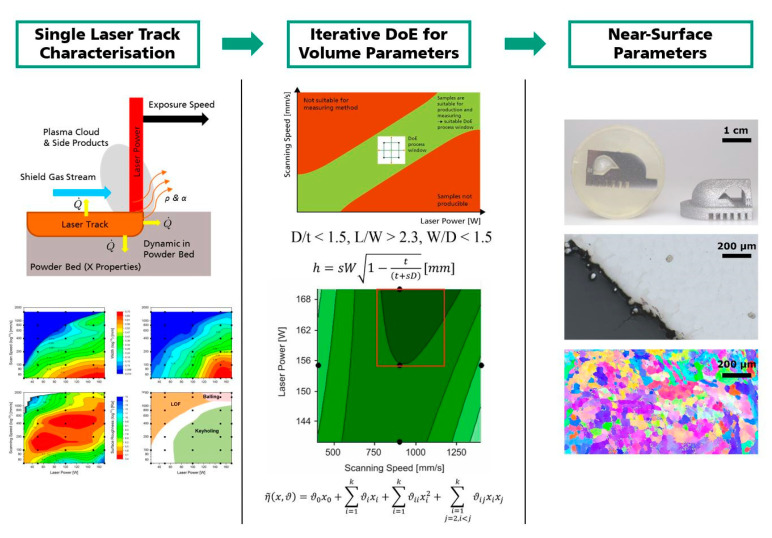
General outline of the approach representing the core steps.

This work proposes a first universal approach in order to develop initial L-PBF process parameters. The approach is not limited to laboratory L-PBF systems, but can also be applied to industrial L-PBF systems exhibiting a lower flexibility regarding the setting of process parameters. A summary of the general outline is presented in Figure 12. Single laser track experiments characterize the interaction between laser beam and used material and provide a fundamental database for the following development steps. The development of volumetric process parameters follows. Thereupon, process windows suitable for a DoE approach can be defined by avoiding areas unsuitable for production or measurement. The iterative use of the DoE approach and the resulting model equations provide information about relevant process windows. Based on this information and defined target values (e.g., maximum relative density) process parameters can be optimized. Near-surface process parameters are developed upon the chosen volumetric parameters and the information gained by the single laser track experiments. A metallographic analysis of a benchmark geometry enables statements regarding the suitability of these locally applied parameters.

A visualization of the complete procedure in its current state is shown in Figure 3. The targeted main application of the methodology is the initial development of process parameters. However, the information generated can also be used to generate component-specific parameters (optimization of material properties and productivity), to optimize existing standard parameter sets with respect to new target values or to structure materials in order to generate designed microstructures. 

The methodology is in permanent development based on the current state of the art. Single steps can potentially be replaced by reliable process simulations. However, since process simulations currently lack reliability and additionally require calibrations and validations, the presented approach is experimental. Further future potential application could implement approaches to transfer parameter sets to other L-PBF systems or layer thicknesses, since parameters can currently not directly be transferred. This can be due to individual machine conditions like the characteristic of the inert gas flow, powder bed heating, recoating system, optical units or laser caustic. In addition, the approach could be adapted to certain material classes in order to encounter challenging problems like micro cracks or poor absorption coefficient.

## Figures and Tables

**Table 1:** The approach was successfully tested for the initial development of process parameters for the following alloys (*t*: layer thickness). Results measured by the Archimedean principle according to DIN-EN-ISO-3369 [38].

Alloy	Achieved Relative Density
Pure Tungsten ^1^ (*t* = 20 µm)	99.51% (σ = 0.018)
Pure Tantalum ^1^ (*t* = 20 µm)	99.99% (σ = 0.022)
MS1 ^1^ (*t* = 30 µm)	99.52% (σ = 0.048)
AlSi10Mg ^2^ (t = 90 µm)	99.18% (σ = 0.091)
Scalmalloy^® 2^ (*t* = 60 µm)	99.69% (σ = 0.197)
1.6947 ^1^ (*t* = 30 µm)	99.55% (σ = 0.056)

^1^ machine type: EOS M100; ^2^ machine type: EOS M400.

**Table 2:** Example for the definition of process parameters for MS1 (*t* = 30 µm). Classifications based on [4,6,7]. p.s. = “per specimen”; p.b. = “per build”; m.s. = “machine setup; u.m. = “used material”; tbd = “to be defined”; PSD = “particle size distribution”.

Parameter		Value	Type
**Class: (1) Laser and Scanning Parameters**			
Mode		Continuous wave	Predefined (m.s.)
Wavelength λ		1070 nm	Predefined (m.s.)
Beam Product		<2	Predefined (m.s.)
Beam quality M^2^		<1.1	Predefined (m.s.)
Intensity profile I		See report	Predefined (m.s.)
Spot size		40 µm	Predefined (m.s.)
Laser power		tbd	Controlled (p.s.)
Scan speed		tbd	Controlled (p.s.)
Hatch spacing		tbd	Controlled (p.s.)
Scan strategy		Stripes and surface	Controlled (p.b.)
Stripes	Stripe width	tbd	Controlled (p.s.)
	Stripe overlap	tbd	Controlled (p.s.)
	Rotation	tbd	Controlled (p.s.)
	Beam Offset stripes	tbd	Controlled (p.s.)
Contour	Laser power	tbd	Controlled (p.s.)
	Scan speed	tbd	Controlled (p.s.)
	Beam Offset Contour	tbd	Controlled (p.s.)
Downskin	Laser power	tbd	Controlled (p.s.)
	Scan speed	tbd	Controlled (p.s.)
	Stripe width	tbd	Controlled (p.s.)
	Beam Offset Donwskin	tbd	Controlled (p.s.)
**Class: (2) Powder Material Properties**			
Alloy		1.2709	Predefined (u.m.)
Particle morphology		Spherical	Predefined (u.m.)
PSD (ASTM-B822)		D10: 17 µm D50: 31 µm D90: 54 µm	Predefined (u.m.)
Pollution		Virgin	Predefined (u.m.)
Tap density		5.2 g/cm^3^	Predefined (u.m.)
**Class: (3) Powder Bed Properties and Recoat Parameters**			
Layer thickness		30 µm	Controlled (p.b.)
Powder bed temperature		Set to room temperature	Controlled (p.b.)
Recoater system		Flexible blade (PU)	Controlled (p.b.)
Recoater speed		0.1 m/s	Controlled (p.b.)
Alloy substrate		Steel (C45/AISI 1045)	Controlled (p.b.)
**Class: (4) Build Environment Parameters**			
Shield Gas		Argon	Predefined (u.m.)
Max. Oxygen		0.1%	Controlled (p.b.)
Overpressure		35 mbar	Predefined (m.s.)
Gas flow		60%	Predefined (m.s.)
Global Offset		tbd	Controlled (p.b.)
Shrinking compensation		tbd	Controlled (p.b.)
